# Transitioning to a life with disability in rural South Africa: A qualitative study

**DOI:** 10.4102/ajod.v10i0.697

**Published:** 2021-07-22

**Authors:** M. Christinah Sadiki, Brian Watermeyer, Nina T. Abrahams

**Affiliations:** 1 Research Administration and Development, University of Limpopo, Polokwane, South Africa; 2 Including Disability in Education in Africa (IDEA) Research Unit, Department of Health and Rehabilitation Sciences, Division of Disability Studies, University of Cape Town, Cape Town, South Africa; 3 Department of Exercise, Nutrition and Health Sciences, Division of Exercise Science and Sports Medicine, University of Cape Town, Cape Town, South Africa

**Keywords:** disability, Global South, rural, qualitative, adjustment, social model, medical model

## Abstract

**Background:**

Adjustment to the onset of disability has complex reverberations relating to both socially engendered disadvantage and the realities of functional limitation. Pre-existing ways of understanding disability can meaningfully shape this experience.

**Objective:**

This study aimed to provide an exploratory understanding of the experience of becoming disabled in a low-income, under-served, rural South African community. In particular, it was interested in how people with disabilities constructed their struggle within the conceptual split between disadvantage caused by ‘malfunctioning’ bodies (a ‘medical model’ view) and that caused by social organisation (a ‘social model’ view).

**Methods:**

Seven people between the ages of 39 and 47 who had acquired a physical disability within the last 4 years were recruited in a rural area of Limpopo province, South Africa. Semi-structured face-to-face interviews were conducted, and the resulting data were thematically analysed. The authors were positioned as both ‘insiders’ and ‘outsiders’ to the participants and sought to use this orientation to best understand and stay faithful to participants’ views while simultaneously applying participant’s experiences to conceptual knowledge in disability studies.

**Results:**

Four themes emerged: (1) emotional impact of onset of disability, (2) being introduced to disablist prejudice, (3) being required to take on a ‘disabled’ identity and (4) socio-economic implications of becoming disabled. The findings reflected a complex set of adverse experiences in the lives of the participants, spanning disadvantages based on embodied, cultural, relational and environmental factors, which were superimposed on existing, generalised poverty in their local communities. Participants made sense of their predicament in multiple, evolving ways.

**Conclusion:**

This study contributes to the understanding of the complex predicaments, and sense-making, of persons who have acquired a disability in a rural, impoverished Global South environment.

## Introduction

Almost everyone will experience a form of impairment or disability (either temporary or permanent) in their lifetime (World Health Organization [WHO] 2011). More than 1 billion people in the world live with some form of disability, of whom nearly 200 million experience considerable difficulties in functioning (WHO 2011). Ageing populations as well as global increases in chronic health conditions such as diabetes, cardiovascular disease, human immunodeficiency virus/acquired immunodeficiency syndrome (HIV/AIDS), cancer and mental health disorders mean that the prevalence of impairments could rise.

In the low-income context of Africa, many people are further disabled by high rates of malnutrition, infectious diseases, violence and injuries and natural disasters, with an estimated 60 million – 80 million people living with disabilities (WHO 2011). People with disabilities are estimated to be 15% of the general population, but possibly higher for those living in poverty (WHO 2011). In the last South African national census, the prevalence of disability was estimated to be 7.5% (Statistics South Africa 2016); however, this may reflect under-reporting because of stigma or limitations of measurement tools (Maart, Amosun & Jelsma 2019).

The World Report on Disability acknowledges disability as a complex, dynamic, multidimensional concept (WHO 2011). In 2001, the International Classification of Functioning, Disability, and Health (ICF) was adopted by the United Nations, defining disability as:

[*A*]n umbrella term for impairments, activity limitations and participation restrictions … Disability is a complex phenomena that is both a problem at the level of a person’s body, and a complex and primarily social phenomena. (WHO 2002:2, 9)

From this grew a body of research examining different understandings of disability, investigating how people from different cultures view the onset of disability, the nature of disability and appropriate interventions for alleviating disadvantage in the lives of people with disabilities (Buntinx & Schalock 2010; Gannotti et al. 2001). Literature from Southern Africa reflects this complexity of experiences of disability. For example, Haihambo and Lightfoot (2010) found that in Namibia, disability is often seen as being caused by supernatural forces, and so is viewed negatively by community members; however, views may change if people are closely connected to a person with a disability. Maart et al. (2019) implemented a survey in two communities in South Africa to demonstrate how disability experiences vary significantly between communities based on the factors such as language, culture and infrastructure.

Given the range of disability experiences, it is important to explore the experiences of people with disabilities in various, and particularly under-researched communities. This article explores these experiences within a low-resourced Global South context. In such circumstances, the onset of disability can compound and complicate existing disadvantage in a host of ways, by adding to the shared community burden of poverty and already limited opportunities for participation in domains such as employment and education (Hosseinpoor et al. 2016).

The site for this study, the Vhembe District of Limpopo province, South Africa, has a largely mining-driven economy with the second lowest gross domestic product per capita out of the country’s nine provinces (Statistics South Africa 2019). The province also achieved the lowest school-leaving certificate pass rate in the country in 2018 and has been shown to have a highly dysfunctional public health system (Mukwevho 2018). A small amount of research on disability has been performed in Limpopo. For example, Luruli, Netshandama and Francis (2016) interviewed people with disabilities attending six rehabilitation centres in the Vhembe District. They found some positive experiences such as good interpersonal relationships with community rehabilitation workers, who adapted to their clients’ needs and provided encouragements. However, there were also negative experiences of poor-quality assistive devices, community barriers towards accessing services and infrequent community worker contact. Other research in disability conducted in this community includes work on the perspectives of physiotherapists working with people with disabilities (Maleka, Franzsen & Stewart 2008), experiences of parents with children with disabilities (Sadiki & Mashegoane 2019), experiences of students with disabilities at universities (Mudau, Netshisaulu & Ncube 2019; Phukubje & Ngoepe 2017) and clinical or prevalence research (Geere, Hunter & Jagals 2010; Hundt, Stuttaford & Ngoma 2004). The current study aimed to give voice to the everyday lives of adults with disabilities in Limpopo, with particular focus on how individuals made sense of experiences of inequality and exclusion when acquiring a new disability.

Given that many disabilities occur as a result of medical conditions, and that people with disabilities have often received treatment in line with the biomedical mode of healthcare practice, disability has often been viewed with both the ‘problem’ and the ‘treatment’ being confined within the individual body (Buntinx & Schalock 2010; Watermeyer & Swartz 2016). Within some non-Western societies, as well as certain faith communities worldwide, understandings of disability are also affected by belief systems that give prominence to such forces as fate and divine punishment which are beyond the reach of human intervention (Shuttleworth & Kasnitz 2005). Pfeiffer et al. (2003) also note that disability has often been viewed as a tragedy, a disgrace, shameful, the result of sin or a punishment from God. People with disabilities are regularly seen as objects of pity or a burden to others.

This article explored these experiences through the prism of the debate regarding medical versus social causality of disability-related disadvantage. Approaches to understanding disability inequality have, in the field of disability studies, been dominated by a binary view, which separates the somatic from the social (Haegele & Hodge 2016; Luruli et al. 2016; Shakespeare 2014). Much disability research in the past has assumed a clear division between struggle caused by bodily reasons and that rooted in social causation (Shakespeare 2006; Watermeyer 2013). The medical model encapsulates the biomedical response to disability with the view that physical impairments alone are responsible for disadvantaging people with disabilities, and that the solution for disability therefore lies in ‘fixing’ individual bodies (Haegele & Hodge 2016; Watermeyer 2013). The social model, on the other hand, asserts that people with disabilities are disadvantaged by social organisation, and that impairment is only an issue in the context of societies that maintain unnecessary barriers to participation (Haegele & Hodge 2016; Watermeyer 2013). However, in feminist disability studies, activists argue that experiences of people with disabilities are grey, complex and cannot be reduced to this binary (Haegele & Hodge 2016; Watermeyer & Swartz 2016). This line of theorising acknowledges the reality of pain, fatigue and bodily ‘limitations’ as real aspects of struggle in the lives of people with disabilities, leading to disadvantage that cannot be addressed by altering social arrangements, while also acknowledging the real effects of discrimination and exclusion caused by society.

While disability activism and research has come a long way from a binary model (Haegele & Hodge 2016; Watermeyer & Swartz 2016; WHO 2002), experience on the ground indicates that people in South Africa may still hold a very medicalised and oppressive view of disability. For example, in South Africa, disability grants are still largely assessed only by medical professionals as opposed to a holistic team (Gathiram 2008). Keikelame and Swartz (2016) interviewed South Africans with epilepsy who reported that healthcare providers were often untrained in disability and considered their patients as just ‘bodies’ without personal stories and needs, leading to inadequate care. Other research in a rural community of Mpumalanga in South Africa indicated that the ICF model and related policies did not accurately capture the experiences of people with disabilities or the barriers that they face and concluded that a focus on context and cultures was integral to improving the lives of people with disabilities (Neille & Penn 2015).

This article builds on PhD research previously conducted on the experiences of newly acquired disability in a South African Limpopo community. This prior study introduced, but did not explore, themes around the role of witchcraft, functional limitation and social exclusion in disability experiences of the study population (Sadiki, Radzilani-Makatu & Zikhali 2018). The present study further develops the theme of the relationship between bodily ‘limitations’ and exclusion in this same study sample. It then uses this theme as a bridge to understand the larger conceptual question about how people with disabilities construct their struggle in relation to the binary conceptual split between disadvantage caused by ‘malfunctioning’ bodies (a ‘medical model’ view) and that caused by social organisation (a ‘social model’ view). We investigate how these two views intertwine in the accounts provided by community members with disabilities, taking cognisance of the influence of resource constraints characteristic of many rural communities in the Global South.

## Methodology

### Research design and data collection

This study was interested in understanding participants’ perspectives on the process of becoming disabled in a previously understudied population. It therefore made use of a qualitative research design that enables one to gain insight into the informants´ life world or lived experience (Creswell 2009; McMillan & Schumacher 2010). Qualitative research is suited to providing thick, detailed descriptions of human experience, which require that researchers recognise themselves as co-constructors of knowledge, which will be in part shaped by their identities, perspectives and personal histories (Denzin & Lincoln 2011). Qualitative research that makes use of interviews is useful in developing a deep understanding of a phenomenon, within its unique set of circumstances (Silverman 2013).

Inclusion criteria included persons who were above the age of 18, spoke Tshivenda and had acquired a physical disability within the 4 years prior to the research taking place (across the PhD period of mid-2010s). Using convenience sampling, all members of the Limpopo Province Disabled People South Africa (DPSA) were sent an invitation to participate by the first author. Seven eligible people agreed to participate. Data were collected through in-depth semi-structured face-to-face interviews. The interviews took place in participants’ homes, at their convenience, and lasted approximately 1 h each, in order to make provision for participant fatigue.

All interviews were tape-recorded, and the interviewer additionally made hand-written notes. Participants were given an opportunity to express themselves in their own words and in their home language of Tshivenda. Voluntary-written consent was obtained from the participants before data collection.

### Participants

Participants in this study comprised seven adults (three men and four women), aged between 39 and 47 years, who had acquired a physical disability in the last 4 years. All lived in the Vhembe District of Limpopo province. Physical impairment, as opposed to other forms of impairment, was chosen as DPSA members largely have physical impairments, and therefore, a greater sample could be expected. It also allowed for greater coherence of the data in shared areas such as access to the built environment and transportation. While any data on an under-researched community such as this one is valuable, it must be acknowledged that the findings cannot necessarily be generalised to other populations such as those that are younger or live outside of the rural Limpopo context. In addition, members of DPSA may perhaps be more educated, have a higher economic status or be more socially connected than other people with disabilities in this context, and the lack of other forms of disabilities represented in this research is a limitation.

### Reflexivity

In making sense of qualitative data, transparency and reflexivity on the part of those collecting and analysing the material is essential (Denzin & Lincoln 2011). The positionality of interviewers, in particular, will have implications for how interviewees respond to questioning, as shared or divergent identities create silences or affordances in what can be said (Gibson & Brown 2009).

In this study, all interviews were performed by the first author, a choice made deliberately based on her sharing aspects of identity, language and local insider knowledge with the participants. The first author is, like all interviewees, a first-language Tshivenda speaker who grew up in a rural area of South Africa. She is a parent of a child with a disability, was at the time working towards a doctorate in disability studies and has extensive experience in disability-related community development initiatives in the area where the research was conducted, including the creation of disability advocacy forums and mentoring groups for mothers of children with disabilities. She worked as a provincial manager for the DPSA for eight years, and consequently, three of the participants knew her and four had heard of her. While the first author was no longer with the DPSA at the time of the interviews, this relationship helped foster trust in that the interviewer was known to be genuinely interested in the experiences of the interviewees. The second author, who performed part of the data analysis and write-up, is a person with a disability, clinical psychologist and disability activist.

The position of the first two authors as both insiders and outsiders to issues in the lived experience of disability was understood as simultaneously potentially advantageous and disadvantageous – serving to enrich understanding of the material through a degree of standpoint identification, while also presenting the risk of excessively subjective interpretations of the data (Gibson & Brown 2009). The first author is an insider in that she shares cultural bonds with the participants and the second author shares the experience of being disabled. The authors were also outsiders in that they hold positions of power by virtue of filling the role of the researcher and having higher education qualifications. However, this dual position of the authors allowed for the application of conceptual thinking in terms of disability theory, as well as a standpoint position that supported a faithfulness to the data.

### Data analysis

The lead author transcribed the gathered information and translated interviews into English for analysis. To analyse and interpret the data, the descriptive analysis technique of Tesch’s eight steps was used (Creswell 2009). This includes identifying categories in the transcripts, clustering these categories according to similarities to themselves and the literature and identifying and grouping themes across the categories.

## Findings

The following four themes emerged: (1) emotional impact of onset of disability, (2) being introduced to disablist prejudice, (3) being required to take on a ‘disabled’ identity and (4) socio-economic implications of becoming disabled. In the following sections, each theme will be discussed, in turn, and illustrated with verbatim quotations from the interview data. Thereafter, a brief discussion and conclusion will be presented.

### Emotional impact of onset of disability

The participants in the study were specifically persons not born with disabilities, but instead had all acquired their disabilities when adults. All reported experiencing great emotional turmoil as a result of the inability to use their bodies in ways they used to, and the loss of the ability to perform the roles expected of them within their physical and social contexts:

‘I could not assume other responsibilities as a mother of the family. My body is not functioning well like before.’ (Participant, C, Female, 42 years old)

The participants stated that, in their understanding, a major source of their pain and problems lay in their impairment, even expressing anger towards their bodies for causing the new struggles that they had to experience:

‘Yes, disability changes life because for me is difficult to adjust, it limits me, and it is like you were once two but now you are one doing the same job. It changes the plans you had for the future and the success you can achieve is reduced.’ (Participant F, Male, 44 years old)

This way in which participants made initial sense of their disabilities seems to fit with the logic of a ‘medical model’ view, which is a highly pervasive way of seeing disability across most societies. Through this lens, disability is reduced to an individual issue wherein individuals are viewed as either a powerless victim or as being responsible for their condition (Baril 2015; Wendell 1997) – a position that often leads to the individual having to take responsibility for no longer being able to participate in society as before (Haegele & Hodge 2016; Watermeyer 2013). In saying this, the authors acknowledge that recognising bodily ‘limitation’ is not in and of itself representative of the full ‘medical model’ view, but that it does carry a logic wherein the body is regarded as the source of disadvantage.

The resultant sense of responsibility may then lead to increased pressure and stress on those with disabilities to adapt and cope:

‘I feel jealous to those who can do things on their own. My physical disability stress; I sometimes feel bitter and neurotic.’ (Participant D, Male, 41 years old)

Evidence has shown how the onset of disability occurs for most people, unsurprisingly, against the backdrop of very limited awareness of how disablist exclusion functions, and the reality that it is all around us (Watermeyer & Gorgens 2013). In such a context, it follows that in these early stages of an acquired disability, one’s understanding of how disadvantage occurs may tend towards ‘medical’ explanations, that is, to most starkly recognising limitations and the impairment. For the participants in this study, a prominent position taken focused on the traumatic and disadvantaging loss of functioning caused by impairment:

‘This is the most painful experience for any human being. It is just unfortunate that you don’t stay with me you would have seen what I am going through.’ (Participant B, Female, 43 years old)

Without any way undermining the reality of bodily limitations that the participants struggled with, a closer examination of the anger directed at ‘malfunctioning’ bodies creates a slightly different picture to the ‘medical model’ views expressed above. The subsequent themes that are explored reveal that feelings of individual limitation and anger directed towards the ‘disabled’ body occur within a cultural context of meanings about what disability is, and what it does.

### Being introduced to disablist prejudice

The study revealed that the process of becoming disabled not only changed participants’ bodies, but also exposed them to new experiences of prejudice and discrimination (Haihambo & Lightfoot 2010; Pfeiffer et al. 2003). This experience has been referred to as ‘a crash course in the harsh realities of social inequality’ where people who become disabled suddenly are explicitly aware of, and receivers of, the prejudice held by some people without disabilities (Watermeyer & Swartz 2016:273).

Morris (2005) considers two common societal attitudes that can, to the extent to which they cannot be resisted, force people with disabilities into a second-class citizen role. The first of these crude stereotypes is that people with disabilities are often assumed to be inherently dependent, like children or animals, in order to complete day-to-day activities. The participants reported experiences of being looked down upon and being patted on the shoulder in a way that is reserved for those in society who are not capable of making their own autonomous decisions:

‘I hate to be treated like a child because of my disability. I have to explain to everyone who help me the cause of my disability and be pitied for [*it*]. [*It*] is like I am paying for the help. “Shame, sorry,” words I came across most of the time.’ (Participant A, Female, 45 years old)

The second common attitude involves people without disabilities at times responding to people with disabilities as if they do not really belong to their society. Some participants reported being called names after acquiring their impairment. For example, *U la munna wa tshihole* means ‘that disabled man’ in Tshivenda, the local language. However, the prefix ‘tshi’ is a derogatory term usually associated with animals and not human beings. The language used by the community to describe the participants illustrates how they were suddenly thrust into a lesser status among their peers without disabilities. From a critical psychoanalytic perspective, such distancing and rejecting attitudes may be understood as motivated by psychological defence mechanisms triggered by the anxieties which disability can evoke (Watermeyer 2006). In particular, people without disabilities may wish to avoid seeing bodies that appear different, or adults who appear dependent, as this may be a reminder of threatening realities to do with the universality of human frailty, and of mortality itself (Watermeyer 2013). In order to create distance from these anxieties, the dominant group in society instead shifts its worst thoughts and fears onto people with disabilities, constructing this group as the personifications of all that is unwanted in the human condition (Watermeyer 2013). Fears that we are unlovable, or incapable are instead then given to those with impairments so that we can feel an illusory sense of relief that ‘it is not us that is broken but them’. This often implicit thought process can manifest in various ways and influences how people interpret disability and interact with individuals with disabilities.

Through experiences like being infantilised, and the language others used to describe disability, participants in the study expressed that they often experienced this implicit and explicit othering from those around them:

‘Sometimes I feel shy because I want to be like able-[bodied] people, to escape negative attitudes.’ (Participant B, Female, 42 years old)

‘Being pitied made me feel disempowered.’ (Participant B, Female, 42 years old)

Distancing attitudes can have various and far-reaching effects in the lives of people with disabilities. Importantly, these attitudes may limit the willingness of communities and governments to provide the resources necessary for individuals to take part in the life of the community, resources that would counteract the effects of the limitations brought on by the impairment itself (Haegele & Hodge 2016; Sen 2000). In line with the social model view, we begin to see how the participants are then not disabled by their impairment but rather, or also, by the exclusionary attitudes of their community.

### Being required to accept a ‘disabled’ identity

The participants reported a variety of domains of social exclusion that were experienced as solidifying their new status as marginal citizens. Three particular sets of exclusionary interactions identified in this study were those with health providers, with the built environment and with family and friends.

#### Health providers

A specific example of societal prejudice experienced by the participants was the prejudice against women with disabilities and their sexuality and reproduction. In this study, it was found that health practitioners stigmatised women with disabilities by disparaging their abilities to be mothers. Two participants revealed the negative attitudes of health practitioners who expressed shock at seeing pregnant women with a physical disability at reproductive health clinics. One participant said that she was made to feel as if she had committed a sin by becoming pregnant:

‘This is my first-born child. I was asked “how will you take care of your child?” Many questions were asked as if I was in court.’ (Participant C, Female, 42 years old)

Such treatment embodies a form of dehumanisation, where participants were regarded as less than full members of the human family, not being entitled to engage in sex and reproduction. Further, the implication was that people with disabilities are not capable of forming and sustaining reciprocal loving relationships. These prejudiced ideas resulted in the view that pregnant women with physical disabilities must have been raped, and that they would not be able to care for a baby (Mgwili & Watermeyer 2006):

‘Shame … shame … Why do you get pregnant out of marriage? You should have used prevention.’ (Participant E, Female, 46 years old)

‘Who will take care of your baby? Who raped you? I was asked embarrassing questions by nurses at my local clinic.’ (Participant C, Female, 42 years old)

Participants reported that the negative experiences and perceptions resulted in them distrusting health practitioners and losing confidence in their services. In the quotations just cited, however, it is noteworthy that in the use of words such as ‘in court’ and ‘embarrassing’, there was a recognition among participants that the treatment they were receiving was inappropriate and discriminatory. Grobbelaar-du Plessis (2007) found that most women face a spectrum of gender-based human rights abuses, but these abuses are magnified for women with disabilities because of their social isolation and presumed dependency. Women with disabilities are also often not regarded by society as being fit to fulfil the traditional roles expected of women, such as mother, wife, homemaker and nurturer (Brodwin & Frederick 2010; Grobbelaar-du Plessis 2007; Mall & Swartz 2012). Even if the participants’ impairments did not impede their ability to have a sexual relationship or give birth to and parent a child, the health providers’ attitudes threatened their receiving of adequate and respectful healthcare.

#### The built environment

In daily life, participants quickly discovered immense restrictions in physically accessing public facilities, such as transport and the built environment. Besides the reality of material exclusion from participation which this brought, it also communicated an implication of no longer being entitled to belong fully in their community:

‘Taxi drivers … are problematic … one day in the morning I waited at the bus stop for two hours because taxis were just passing when I stopped them. I was told to close the door because there is no space for a wheelchair.’ (Participant C, Female, 42 years old)

‘My capabilities and opportunities are being restricted by an inaccessible environment.’ (Participant E, Female, 46 years old)

The participants reported feeling angry and distressed that they were denied the opportunity to participate equally as they had before. Again, there was ambiguity in their views regarding which part of their disadvantage was socially engendered, and which was an unavoidable consequence of a bodily ‘limitation’:

‘I remember the other day I visited my nearest local clinic to collect some medication. It happened that I had to use the toilet. When I got there it was a disaster because the door was too narrow for my wheelchair to get in and I was very pressed. I was very upset with everyone and I did not have any choice but to go back home … by the time I arrived home, I had already messed-up … You know I won’t forget that day. My sister, tell me how will you feel if it was you?’ (Participant B, Female, 43 years old)

Exclusion resulting from inaccessible built environments and services not only disadvantages in material, tangible terms, but, as noted above, can also have a malignant effect on emotional well-being, through causing an understandable withdrawal from what are experienced as unwelcoming public spaces. In addition, the lack of usable services can create dependence on friends and family, which may have the effect of confirming harmful and prejudiced stereotypes, implying that people with disabilities are ‘in fact’ not capable of real participation.

#### Friends and family

Alienating responses from the broader community were also experienced in interactions with family and friends. Participants reported that their disability put new forms of strain on relationships with family members. This was attributed to increased levels of physical care and support needs relating to their impairment. This is particularly pertinent in a low-resource setting, where there is a lack of availability and accessibility of paid professional assistance for people with disabilities (WHO 2002). What appears significant here is how dependency needs, which as noted are often based on unnecessary inaccessibility of environments and services, can distort even long-standing relationships. Watermeyer and Swartz (2008) note how the need for care can confuse relational boundaries, creating a situation in which people with disabilities may feel obligated to remain grateful, patient and flexible, even in the face of very difficult circumstances.

In the study, participants described feeling ashamed and self-conscious about their need for assistance, a position from which it is likely to be difficult to express one’s feelings and needs clearly. This can have important implications for self-advocacy, and the articulation of experiences of prejudice or marginalisation:

‘For me I feel as a burden to my household members. I constantly need help from others.’ (Participant E, Female, 46 years old)

The authors found that there can be extreme emotional vulnerability at play in circumstances where one, as a person with a disability, requires daily assistance with intimate aspects of life, while at the same time is aware of devaluing attitudes, or even disgust, held by the person who one is assisted by. For example, some participants experienced openly prejudiced responses from family members who were embarrassed by their presence. Painfully, one participant told of how family members discouraged him from attending a family funeral, placing the comment in the guise of it relieving all from a difficult physical challenge:

‘It is sad … can you believe that my family refuse me to attend family and community funerals because no one will have time to assist me and everyone will need to know about my disabilities.’ (Participant G, Male, 41 years old)

This experience felt profoundly rejecting and contributed to the sense of isolation and non-belonging felt by the participant because of prejudice, rather than simply impairment.

On an ongoing basis, participants reported that their disability resulted in diminished quality contact with family and friends. The inaccessible environment, in combination with attitudes towards disabilities, meant that while these participants were not necessarily purposefully ‘hidden’ from the community, they were often restricted to their homes and isolated from mainstream society:

‘… [*Disability*] affected my life as all the people I used to socialise with are no more there; I had to start to create new friends who understood my new condition.’ (Participant C, Female, 42 years old)

These experiences of altered friendships, the emergence of prejudiced attitudes and the withdrawal of support created a great sense of distress among participants. However, they often felt restricted in their ability to express these feelings to those around them as they were expected instead to be grateful for the care they did receive and not to be a burden to others:

‘I do appreciate for the support. I always make sure that I don’t keep on asking for help because I don’t want to upset anyone because I depend on their support, sometimes I feel as if I am requesting too much and I don’t want to spoil friendship to anyone.’ (Participant A, Female, 45 years old)

The socially engendered constraints maintained over the expression of negative feelings, or objections to exclusion, by people with disabilities, is a well-established issue in disability studies (Thomas 1999; Watermeyer 2009; Watermeyer & Swartz 2008). This presence within participants of feelings of rejection resulted at times in instances of voluntary isolation to avoid these feelings being displayed:

‘Most of the time I avoid visiting public places and opt to stay home to avoid bothering other people for asking for help.’ (Participant B, Female, 43 years old)

Evident here is the predicament of people with disabilities living in inaccessible environments, who are thus forced to rely on assistance from friends and family, potentially harming relationships, and carrying implications for power inequality (Watermeyer & Swartz 2008).

### Socio-economic implications of becoming disabled

It is known that people with disabilities are more likely to be unemployed and experience higher rates of poverty with fewer resilience options than their peers without disabilities (Wapling 2012). Disability and poverty have been shown to interact in complex ways, where functional difficulties may result in reduced productivity, causing higher susceptibility to poverty, while poverty reduces a person’s access to assistive devices, accessible infrastructure and rehabilitation services that could support economic participation (Maart et al. 2019; Mitra 2018; WHO 2002). In line with this, since becoming disabled, some participants lost their jobs and sources of income:

‘Lack of source of income is a challenge because I am unable to meet the family responsibilities as I used to before acquiring the physical disability.’ (Participant F, Male, 44 years old)

‘It is not easy; the disability grant is the only source of income for my family.’ (Participant D, Male, 41 years old)

In South Africa, all persons with disabilities are theoretically eligible for a disability grant; however, only a small percentage receive one in practice (Gathiram 2008). All the participants reported that they receive financial support through a disability grant from the government which reflects their (relatively) ‘privileged’ position in the disabled community. However, even with the grant, they were unable to sustain their families. This problem is especially acute in a low-income setting where all family members may be struggling economically, even if non-disabled. Placing further economic strain on the family, participants were firstly unable to contribute to the household via employed work, and secondly, reduced the labour power of the household through their need for care from others. One participant explained that her mother was no longer able to work in order to be available to assist her:

‘My mother was a domestic worker, but due to my physical disability, she could not continue with her job.’ (Participant E, Female, 46 years old)

The authors considered that this may place further strain on relationships, as economic stress may result in implicit or overt resentment of the needs and dependency of the family member with a disability. Paradoxically, family members in poor households may also be dependent exclusively on the social grant of the family member with a disability for essential items such as food:

‘This Government has made access to disability grant. I am currently receiving disability grant which I receive every month from the Government is for me to feed my family and that is the only income I receive in my life unfortunately.’ (Participant G, Male, 41 years old)

One possible unintended effect of being a recipient of social support programmes is that people with disabilities may be viewed as incompetent, as they are unable to make ends meet even though they are seen as already receiving government support (Tronto 2010). Family members with a disability may even be resented for receiving ‘free’ money from the government, further undermining their ability to ask for the practical help they needed:

‘Yesterday I went to my cousins’ house with a 10 kg container to ask for [*maize meal*]. His wife said bad things to me, she wondered why I could not afford to buy just a mere bag of [*maize meal*] when I am receiving a disability grant from the government every month.’ (Participant C, Female, 42 years old)

‘My grandmother also talks too much when I go and ask her for food. Everyone will know that I did ask her for food, so it is better not to go and ask her for food because she cannot keep it to herself. I am pulling hard and I don’t want everybody to know about my difficulties.’ (Participant B, Female, 43 years old)

A further aspect of life on a social support grant experienced by participants was a belief that they should be content – grateful – that they can survive off social welfare, and thus do not need or deserve to be accommodated as full economic citizens, further limiting their opportunities to engage in the community (Surender et al. 2010):

‘My uncle told me not to stress about getting job because I am getting disability grant which I can use it to buy food. I am stressed for not getting a job because I have family to take care of. Disability grant is not enough for my basic needs, people think that disability grant to persons with disabilities is a lot of money. We are human beings like others and have responsibilities too.’ (Participant A, Female, 45 years old)

‘In 2011 there was a community project in my village and community members were priorities for employment. I submitted my personal details to the community leader on time. After two weeks the project started, and I was not called to start working with others. When I make follow up to the project leader, I was told that I must give chance to other community members because I am getting disability grant.’ (Participant A, Female, 45 years old)

Research from South Africa indicates that while grants may offer temporary relief, if people with disabilities are unable to access employment and education, they will continue to be marginalised from society and continue to be dependent on government support (Loeb et al. 2008). This vulnerability is exacerbated by the fact that in rural areas of South Africa such as those where the study took place, there are few resources to provide services, roads and infrastructure for the general population, further leaving persons with disabilities behind (Mitra 2018).

## Discussion

Participants in this study experienced complex changes to their bodies, their relationships, their environment and their economic participation after becoming disabled in adulthood. Their stories reflected an intimate intertwining of aspects of the disability experience stretching from impairment effects to material barriers to participation, as well as the harmful, even corrosive presence of psycho-emotional disablism within the community (Morris 2005; Thomas 1999; Watermeyer 2013). As experienced by the participants, it is not only broader society but also family and friends who often hold explicit and implicit attitudes of rejection and shame towards a person with a disability (Friedman 2019; Parr 2007). These attitudes may reflect the broader societal response towards disability, where people without disabilities may overprotect, pity or reject those with disabilities to insulate their own identities from the stigma of ‘broken’ bodies (Watermeyer & Swartz 2008) and often do this by over-medicalising people with disabilities and putting responsibility on them to adapt to society (Haegele & Hodge 2016; Neille & Penn 2015). This can, and for the participants it did, result in extremely painful experiences of alienation and even dehumanisation.

For the participants in this study, becoming visibly disabled meant coming up against previously invisible forces, which served to push them towards a marginal social position. The participants at times expressed deep feelings of emotional turmoil and hopelessness regarding their impairments, citing their disability as painful and limiting. This reflects an understanding of disadvantage being something which emanates from the body – a view that leans towards a medical model logic. However, throughout their accounts, this ‘medicalising’ sense-making was intermingled with stark recognition of barriers to participation as incidences of social injustice, amid the growing realisation of their new positioning as marginal. Physical barriers reported by the participants further contributed to feelings of alienation, as they found themselves in a new world containing multiple levels of exclusion. Some of the clearest incidences of this recognition were caused by the nature of the built environment and transport services, as well as the responses of reproductive health practitioners to women with disabilities. These effects were enacted both interpersonally and structurally, superimposed on the emotional trauma which all described as part of the onset of functional limitation.

The authors acknowledge that this is a modest study with a small sample size. In addition, the sample may be relatively ‘privileged’ compared to poorer or less connected people with physical disabilities or other less recognised disabilities in this community – who may experience even more marginalisation. However, we believe that the data provide accounts that demonstrate meaningful links between lived disability experience and conceptual understandings. If we consider the experiences and sense-making of the participants in this study in terms of the three disability paradigms described earlier – that is, the medical model, social model and feminist views – we find that the data reflect an interweaving of all three perspectives. While most participants described having attributed exclusion to their impairment within the first few years of acquiring a disability, this view fluctuated and changed in response to different life experiences, to include greater, and even vehement, recognition of the reality of material barriers to participation. However, taking this increasingly social model-oriented view did not limit recognition of neither relational aspects of disablism (Reeve 2006; Watermeyer 2013), nor the reality of functional limitation because of impairment. In this, the participants’ understanding of their social and embodied circumstances mirrored a feminist view, which augments the social model with aspects of life with disability, which exist in the private realm, are relational as well as structural, and are not amenable to binary logic (Shakespeare 2006, 2014; Thomas 1999). In this way, the accounts gathered in this study showed how views demonstrating both social model and medical model logics served to illuminate relevant aspects of participants’ experience in a complementary manner, rather than constituting mutually exclusive positions. Further, while not the only framework relevant to exploring disability experiences, the feminist perspective proved useful in illuminating other aspects of the participants’ accounts, making provision for the lived realities of power, oppression and disadvantage starkly seen in poorer communities (Hall 2015). These realities are important to understand in order to make meaningful policy and cultural shifts towards disability inclusion. (Neille & Penn 2015).

As an experienced government official working in community-based disability development in the area, and a mother of a child with a disability, the first author’s own reflections revealed that she brought hope and expectation to the study that participants would have received more community support, and that they would have understood their experience of disadvantage as originating mainly in the environment rather than the nature of their bodies. However, the author had to acknowledge that this viewpoint may have been influenced by her educational and employment background, and that adequate support and disability activism, bringing emancipatory understandings of disability, may not be readily available in this rural context. Acknowledging this ‘outsider’ perspective encouraged the author to reflect and report on the full experiences of the participants, even if painful and indicative of a gap in what she was able to provide while working in disability support in this community.

In 2007, South Africa ratified the United Nations Convention on the Rights of Persons with Disabilities which enshrine principles of inclusion and equity for people with disabilities in all spheres of society (Swanepoel 2020). However, in the Global South context of rural South Africa in which poverty and a lack of social services is a common problem, the participants in this study experienced the disadvantaging effects of shaming attitudes, over-medicalisation and a lack of appropriate impairment-related assistance, threatening to cement harmful stereotypes about both the dependency of people with disabilities and their entrapment in poverty. Put another way, what was in evidence were environmental factors which threatened to bring the most harmful stereotypes about rural life with disability into reality, along with victim-blaming ‘medical model’ logic. This was further confused in this rural setting, where participants may be both marginalised by physical and attitudinal barriers, and perceived as receiving ‘special treatment’ via disability grants.

For anyone, the experience of having to struggle to survive and access basic resources such as food embodies an attack on dignity. However, when one has essential support withheld, and must also face denigrating attitudes from others, people with disabilities describe how hard it can be to hold onto a clear sense that one is as deserving as anyone of having their rights to inclusion fulfilled (Reeve 2006; Thomas 1999; Watermeyer 2013, 2016). While the limitations caused by impairment are real and have real effects, the attitudes that fuelled social interactions and the rural setting that contributed to inaccessible environments greatly exacerbated the disability experiences of the participants in this study.

## Conclusion

This study, we believe, has gone some way to exploring the ways in which rural South Africans make sense of their experience of physical disability, and how this sense-making fluctuates and moves between impairment-based and socially orientated meanings. It shows a complex and intertwined experience of multiple barriers that are relational, embodied, material and structural, which together can affect one’s ability to feel fully accepted by their community. The nuanced meaning-making revealed in the data shows up the benefits of an analysis of disability disadvantage which is not caught in binary logic. Indeed, over the past two decades, disability studies have seen an integration of approaches that previously might have been marked out in opposition to one another, as ‘medical’ versus ‘social’ model views (Haegele & Hodge 2016; Shakespeare 2014). The body and psyche are very much part of the stories of disadvantage being told in this study, echoing the affective turn in the social sciences more generally (Watermeyer 2013). But the constraining reality of structural disadvantage, delimiting not only what one may access, but also who one is able to be, is just as evident.

At the time of onset of an impairment, people need social support perhaps more than at any other time in their lives. Instead, the participants in this study experienced a profound destabilising of many of the foundations that afford any community member a sense of security and belonging. The sample size in the study was small, and included persons living with only one form of impairment, both of which are limitations. Nevertheless, we believe that experiential data help to give voice to the multi-level complexity of socially engendered disadvantage surrounding disability, which is of relevance to the designing of interventions that are effective and locally relevant. It is desired that this article will stimulate future research to further contribute to our shared understanding of the predicaments of people in rural, Global South contexts, who acquire disability against the backdrop of ongoing, generalised poverty, in support of a more informed response from both government and community sectors.
